# Up-regulation of ABCG1 is associated with methotrexate resistance in acute lymphoblastic leukemia cells

**DOI:** 10.3389/fphar.2023.1331687

**Published:** 2024-01-08

**Authors:** Yao Chen, Houshun Fang, Huiying Sun, Xiaoyu Wu, Yan Xu, Bin-Bing S. Zhou, Hui Li

**Affiliations:** ^1^ Pediatric Translational Medicine Institute, Key Laboratory of Pediatric Hematology and Oncology Ministry of Health, Shanghai Children’s Medical Center, Shanghai Jiao Tong University School of Medicine, Shanghai, China; ^2^ Fujian Children’s Hospital, Fujian Branch of Shanghai Children’s Medical Center Affiliated to Shanghai Jiaotong University School of Medicine, Fuzhou, China; ^3^ Department of Pharmacology and Chemical Biology, School of Basic Medicine and Shanghai Collaborative Innovation Center for Translational Medicine Ministry of Education, Shanghai Jiao Tong University School of Medicine, Shanghai, China

**Keywords:** ALL, MTX resistance, chemosensitivity, ABCG1, epigenetic modification

## Abstract

Acute lymphoblastic leukemia (ALL) is a prevalent hematologic malignancy in children, and methotrexate (MTX) is a widely employed curative treatment. Despite its common use, clinical resistance to MTX is frequently encountered. In this study, an MTX-resistant cell line (Reh-MTXR) was established through a stepwise selection process from the ALL cell line Reh. Comparative analysis revealed that Reh-MTXR cells exhibited resistance to MTX in contrast to the parental Reh cells. RNA-seq analysis identified an upregulation of ATP-binding cassette transporter G1 (ABCG1) in Reh-MTXR cells. Knockdown of ABCG1 in Reh-MTXR cells reversed the MTX-resistant phenotype, while overexpression of ABCG1 in Reh cells conferred resistance to MTX. Mechanistically, the heightened expression of ABCG1 accelerated MTX efflux, leading to a reduced accumulation of MTX polyglutamated metabolites. Notably, the ABCG1 inhibitor benzamil effectively sensitized Reh-MTXR cells to MTX treatment. Moreover, the observed upregulation of ABCG1 in Reh-MTXR cells was not induced by alterations in DNA methylation or histone acetylation. This study provides insight into the mechanistic basis of MTX resistance in ALL and also suggests a potential therapeutic approach for MTX-resistant ALL in the future.

## 1 Introduction

Acute lymphoblastic leukemia (ALL) constitutes over 25% of childhood malignancies (Malard and Mohty, 2020). Despite the survival rate for ALL exceeding 80% with improved chemotherapy, relapse remains a leading cause of fatality in childhood cancers (Hunger and Mullighan, 2015; Teachey and Pui, 2019).

Methotrexate (MTX) is a crucial chemotherapeutic drug in ALL treatment (Pui and Evans, 2006). As a folate antimetabolite, MTX competitively inhibits enzymes involved in folate metabolism, causing DNA damage and ultimately leading to cell death (Gonen and Assaraf, 2012). Unfortunately, clinical resistance to MTX is frequently observed (Wojtuszkiewicz et al., 2015). Cells uptake MTX through the reduced folate carrier (RFC1) (Hou and Matherly, 2014). Once MTX enters the cytoplasm, polyglutamylation, catalyzed by polyglutamate synthetase (FPGS), occurs (Raz et al., 2016). Polyglutamylation not only retains MTX within the cell but also enhances the inhibition of target enzymes, including dihydrofolate reductase (DHFR), thymidylate synthase (TS), and some enzymes involved in purine synthesis (Assaraf, 2007; Raz et al., 2016). Conversely, γ-glutamyl hydrolase (GGH) hydrolyzes polyglutamate tails to generate the mono-glutamate form, increasing MTX efflux. This efflux is governed by membrane transporters of the ATP-binding cassette (ABC) family, including MRPs/ABCCs and BCRP/ABCG2 (Assaraf, 2006).

Alterations in any step of the MTX metabolic pathway can result in cellular resistance. Over the past few years, various mechanisms contributing to MTX resistance have been reported, such as a) impaired uptake due to inactivating mutations or downregulation in RFC1 (Rothem et al., 2003; Rothem et al., 2004; Kaufman et al., 2006); b) loss of FPGS function due to relapse-specific mutations (Li et al., 2020; Yu et al., 2020); c) enhanced GGH activity or expression (Cole et al., 2001; Schneider and Ryan, 2006; Kim et al., 2013; Wang et al., 2022); d) overexpression of DHRF (Assaraf, 2007; Fotoohi and Albertioni, 2008); e) augmented drug efflux due to increased expression of the transporters such as ABCB1, ABCC1-4 and ABCG2 (Volk et al., 2002; Ansari et al., 2009; Liu et al., 2018; Jaramillo et al., 2019; Engle and Kumar, 2022). It is worth mentioning that the mechanisms underlying clinical resistance to MTX involve multiple factors and are not fully understood.

Aiming to investigate the mechanisms of MTX resistance in ALL, we established an MTX-resistant cell line (Reh-MTXR) in ALL cell line Reh by stepwise selection. We observed upregulation of ABCG1 in the Reh-MTXR cell line. ABCG1, a member of the ABC transporter family, regulates cellular cholesterol and phospholipid homeostasis (Zeng et al., 2023). Dysregulation of ABCG1 has been implicated in various diseases, including cardiovascular disease and diabetes (Sturek et al., 2010). Additionally, high ABCG1 expression has been associated with poor outcomes in various tumors (Wang et al., 2016; Tian et al., 2017; Meng et al., 2021; Xi et al., 2022). However, to the best of our knowledge, so far, the role of ABCG1 in ALL has yet to be reported and explored. Epigenetic modifications have been linked to increased drug efflux pump expression. For example, elevated ABCB1 expression is mediated by DNA methylation and histone acetylation at the ABCB1 promoter (Wilting and Dannenberg, 2012). Similarly, high expression of ABCG2 and ABCC6 is modulated by alterations in DNA methylation (Adhikari et al., 2022). Nevertheless, the potential mechanisms underlying MTX-treatment-induced ABCG1 upregulation remain unclear.

This study demonstrates the upregulation of ABCG1 in MTX-resistant cells. The reversal of drug resistance upon ABCG1 knockdown suggests its functions to MTX resistance. Mechanistically, increased ABCG1 expression promotes MTX efflux, leading to impaired accumulation of MTX polyglutamated metabolites. Moreover, the ABCG1 inhibitor benzamil effectively sensitizes Reh-MTXR cells to MTX. In addition, the upregulation of ABCG1 in Reh-MTXR cells is not associated with epigenetic alterations in DNA methylation or histone acetylation. Our findings reveal a novel chemoresistance mechanism and propose a potential therapeutic target for MTX-resistant ALL.

## 2 Materials and methods

### 2.1 Cell lines and cell culture

The parental cell line Reh and resistant cell line Reh-MTXR were cultured in RPMI-1640 medium supplemented with 10% FBS. The MTX-resistant subline Reh-MTXR was established as follows: parental Reh cells were pulse exposed to 200 nM MTX for 24 h, cells were washed once by PBS, and continued to grow for one to 2 weeks in the culture medium, this process was repeated for a total of 10 cycles. All cells were examined for *mycoplasma* contamination.

### 2.2 Generation of knockdown and overexpressed cell lines

Gene knockdown assays were conducted using lentiviral shRNAs or the CRISPR/Cas9 system. The shRNAs were cloned into the pLKO.1 plasmid (Addgene, Watertown, United States of America), and the shRNA sequences for ABCG1 knockdown were as follows: shABCG1-1, 5’-GAA​GTT​CAA​TAG​TGG​TGA​GTT-3’; shABCG1-2, 5’-CTT​GTG​CCA​TAT​TTG​AGG​GAT-3’. CRISPRs were designed based on information available at http://crispr.mit.edu and cloned into the pLenti-CRISPR v2 plasmid (Addgene, Watertown, United States of America). The sgRNA sequence for ABCG1 was 5’- ACT​GAG​ACG​GAC​CTG​CTG​AA-3’. Transfections were performed using jetPRIME (Polyplus-transfection, Illkirch, France) following the manufacturer’s instructions. Stable knockdown cells were selected with 1 μg/mL puromycin (Thermo Fisher, Carlsbad, United States of America).

The coding region of the ABCG1 gene was cloned into the pSB-Flag vector (Zheng et al., 2023) and confirmed by DNA sequencing. Reh cells were transfected with the ABCG1 expression vector using 4D-Nucleofector (Lonza, Basel, Switzerland), and stable expression was achieved by selection with 1 μg/mL puromycin.

### 2.3 Cell viability assay

Cells were plated in 96-well plates (12,000 cells per well) and treated with a gradient concentration of drugs for either 72 h or 6 h (MTX). In the MTX drug sensitivity assay, after 6 h of MTX exposure, cells were washed twice with PBS and cultured in drug-free medium for an additional 66 h. Cell viability after 72 h was determined using CTG (CellTiter-Glo Luminescent kit, Promega, Madison, United States of America) following the manufacturer’s guidelines. The IC_50_ value, representing the concentration inhibiting 50% of growth, was calculated with GraphPad Prism (GraphPad Software, San Diego, United States of America). For the growth curve assay, cells were seeded in 96-well plates (10,000 cells per well), and daily monitoring of cell viability was performed from day 0 to day 4. The relative growth rate at the indicated time points was calculated with day 0 set as the control.

### 2.4 Apoptosis analysis

Cells were seeded in triplicated in 24-well plates and treated with 2 μM MTX for 6 h. Then, the content was washed once and allowed to continue to culture in the drug-free medium for 48 h or 72 h. Subsequently, the cell samples were stained with annexin V-APC and PI (Elabsciences, Wuhan, China) and detected by flow cytometry (BD Biosciences, New Jersey, United States of America), the percentage of apoptotic cells was analyzed by FlowJo software (BD Bioscience).

### 2.5 RNA-sequencing (RNA-seq) analysis

Total cellular RNA was isolated using the Trizol extraction method (Thermo Fisher, Carlsbad, United States of America). Subsequently, 10 ng–100 ng of RNA was utilized for preparing sequencing libraries with the TruSeq RNA Sample Preparation Kit v2 (Illumina). All samples were sequenced on the Illumina platform Hiseq X Ten with paired-end 101bp reads. To analyze the data, the sequencing reads were mapped to the human reference genome (GRCh37) using STAR (2.7.1a). HTSeq count was employed to calculate read counts for each gene, and fragments per kilobase of an exon model per million (FPKM) were calculated to quantify gene expression levels. Differential gene expression analysis was performed using the DESeq2 package in R. Genes with |log2 (fold change)| > 2 were considered differentially expressed between the two conditions for each group.

### 2.6 RNA extraction and quantitative real-time PCR

Total cellular RNA was obtained by RNA Isolation Kit V2 (Vazyme, Shanghai, China). One μg of the isolated RNA was then reverse-transcribed into cDNA using a cDNA synthesis kit (YEASEN, Shanghai, China). Real-time qPCR was performed using a SYBR kit (YEASEN, Shanghai, China) by a real-time PCR thermocycler (Bio-Rad, Hercules, United States of America). Primers used for Q-PCR were as follows:

**Table:** 

Gene	Forward (5'-3′)	Reverse (5′-3′)
Actin	CAT​CCG​CAA​AGA​CCT​GTA​CG	CCT​GCT​TGC​TGA​TCC​ACA​TC
FPGS	GGG​TGA​CCC​TCA​GAC​ACA​GT	GTC​TTC​AGG​CCA​TAG​CTT​CG
GGH	CTG​CAG​GTG​CGA​GAG​TTG​TA	ACT​TCC​TCC​AGG​GAA​AAG​GA
DHFR	CTC​AAG​GAA​CCT​CCA​CAA​GG	GTT​TAA​GAT​GGC​CTG​GGT​GA
RFC1	CCC​CTC​CAG​CTA​TGA​ATG​AA	GGG​CCC​ATT​TTG​ATA​TCC​TT
ABCG1	ACG​CAG​TTC​TGC​ATC​CTC​TT	CGG​AGT​TGC​TCA​AGA​CCT​TC
ABCG2	AGC​TGC​AAG​GAA​AGA​TCC​AA	TGC​CCA​TCA​CAA​CAT​CAT​CT
ABCC1	CAG​ACT​GGC​AGG​GCT​ACT​TC	CTG​ACG​AAG​CAG​ATG​TGG​AA
ABCC2	AGA​GCT​GGC​CCT​TGT​ACT​CA	TGC​CTA​GGT​AGA​GGC​TTG​GA
ABCC3	TGT​GCT​AGC​TGA​TGG​ACA​GG	GCA​GAG​AAA​GTT​GGC​AAA​GG
ABCC4	GGT​TCC​CCT​TGG​AAT​CAT​TT	ATC​CTG​GTG​TGC​ATC​AAA​CA
ABCC5	AGC​TGA​AGC​GTC​TGG​ACA​AT	GTA​TGC​TGG​ACG​TGA​TGT​GG
ABCB1	CAG​AGG​GGA​TGG​TCA​GTG​TT	CCT​GAC​TCA​CCA​CAC​CAA​TG

### 2.7 Western blotting

Cells were harvested and lysed in LDS Sample buffer. The samples were boiled at 100°C for 10 min and then loaded onto 4%–12% FuturePAGE (ACE, Changzhou, China), followed by blotting onto a nitrocellulose membrane. The primary antibodies included β-actin (66009-1-Ig, Proteintech, Wuhan, China, 1:8000 dilution), ABCG1 (13578-1-AP, Proteintech, Wuhan, China, 1:5000 dilution), FPGS (ab184564, Abcam, Cambridge, United Kingdom, 1:1000 dilution), GGH (13264-1-AP, Proteintech, Wuhan, China, 1:2000 dilution). Signal form immunoblots were evaluated using the Imaging System (Bio-Rad, Hercules, United States of America).

### 2.8 Preparation of metabolite extraction

To induce MTX polyglutamylation, cells were cultured in growth medium containing 2 μM MTX for 24 h. Following the incubation, rapidly washed 1 × 10^7^cells three times with ice-cold PBS and lysed with 1 mL 80% cold methanol. The lysates were then vortexed at 4°C for 30 min and subsequently centrifuged at 20,000 g for 15 min at 4°C to remove cell debris. The supernatants were collected and analyzed by Liquid Chromatograph Mass Spectrometer (LC-MS).

In the investigation of MTX efflux, cells were exposed to 10 μM MTX for 20 min, followed by three washes with ice-cold PBS. Efflux was tracked by replacing the drug solution with drug-free medium, observing efflux at 0, 5, 15, and 30 min at 37°C. Subsequently, 1×10^7^ cells were washed with ice-cold PBS, lysed in 1 mL 80% cold methanol, and the supernatants were collected for analysis using LC-MS.

### 2.9 Liquid chromatographic and mass spectrometric analysis

The LC-MS was performed as previously described (Wang et al., 2022). The standards of MTX-Glu2, MTX-Glu3, MTX-Glu4, and MTX-Glu5 were purchased from Schircks Laboratories (Bauma, Switzerland).

### 2.10 Mouse xenograft models

Xenograft models of human ALL were established in B-NDG (NOD.CB17-*Prkdc^scid^Il2rg^tm1^*/Bcgen). Reh, Reh-MTXR and MTXR-shABCG1 cells (1 × 10^7^ cells/mouse) were injected into the tail vein of NDG mice aged 8 weeks. After 10 days, mice were treated with MTX (10 mg/kg every 3 days for 3 doses). Mice were sacrificed at approximately 3 weeks, and we isolated their bone marrow cells from the tibia bone. Then, we analyzed CD19-positive cells by flow cytometry.

### 2.11 Statistical analysis

Statistical analysis was conducted utilizing GraphPad Prism 8.0 Software (GraphPad Software, San Diego, United States of America). The data were represented as means ± SD obtained from three separate experiments. Two-tailed Student's t-tests were employed for comparisons between the two groups. *p < 0.05, **p < 0.01, ***p < 0.005, ****p < 0.0001 were considered statistically significant.

## 3 Results

### 3.1 Establishment of the MTX resistance cell line Reh-MTXR

**FIGURE 1:**
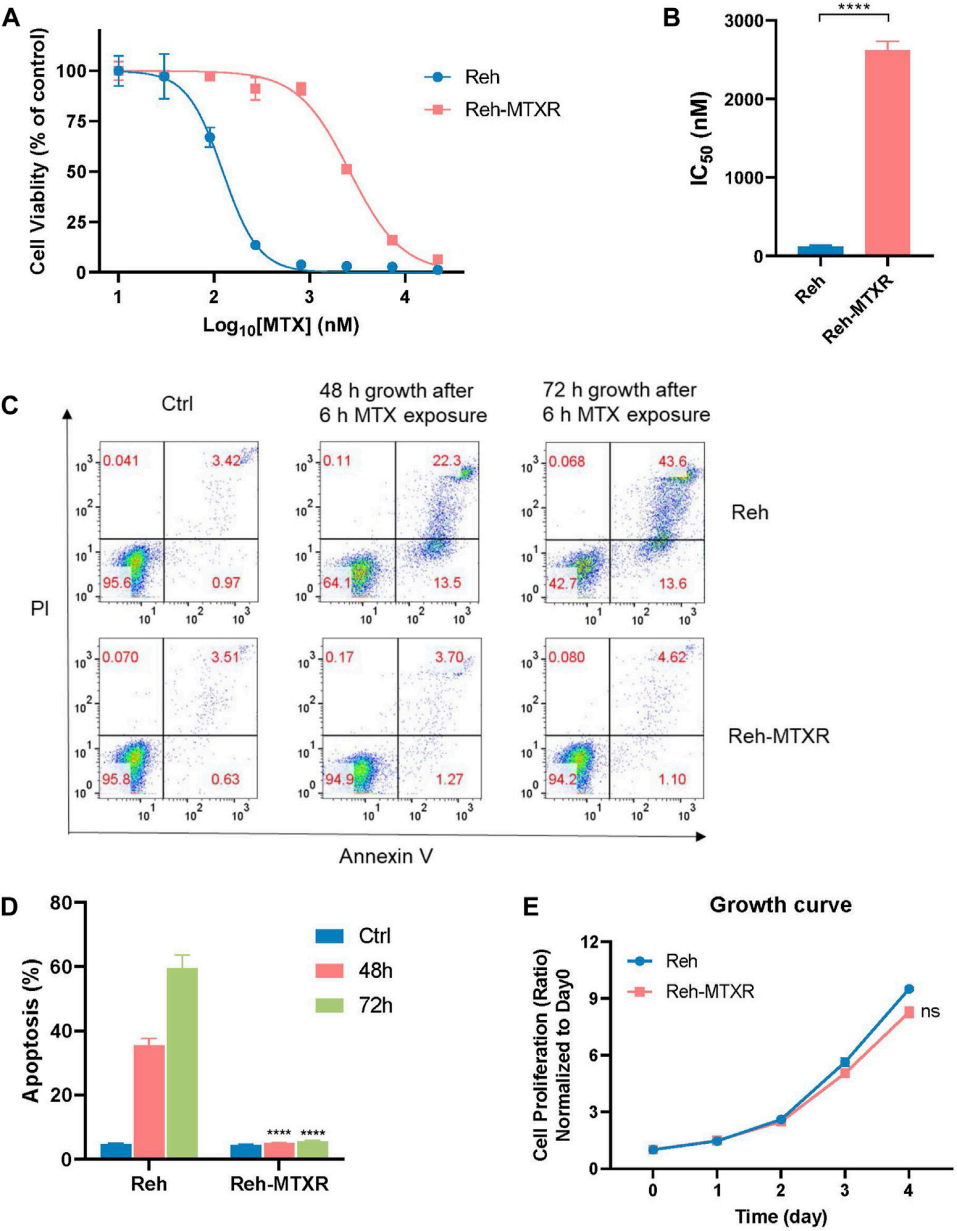
Establishment of the MTX resistance cell line Reh-MTXR. **(A, B)** Reh and Reh-MTXR cells were exposed to increasing concentrations of MTX for 6 h and then incubated in the drug-free medium for an additional 66 h, after which cell viability was determined using the CellTiterGlo assay. **(A)** Cell viability changes are shown as dose-dependent curves. Data are presented as mean ± SD of three independent tests and are indicated as a percentage of the control group, which was treated with vehicle only and set as the 100% control. **(B)** IC_50_ values were obtained from experiments performed as in Figure 1A. *p < 0.05, **p < 0.01, ****p < 0.0001, two-tailed Student’s t-test. **(C, D)** Reh and Reh-MTXR cells were treated with 2 μM MTX for 6 h, and cells were then washed with PBS and culture in the drug-free medium for an additional 48 h or 72 h. Apoptosis induced by MTX was analyzed using a flow cytometry assay. Data are presented as mean ± SD of three independent tests, ****p < 0.0001, two-tailed Student’s t-test, compared with the Reh parental cell group. **(E)** Growth curves of Reh and Reh-MTXR cells grown in RPMI 1640 medium. We calculated the relative growth rate at different time points with day 0 as the control. Data are presented as mean ± SD of three independent tests.

To investigate resistance mechanisms to MTX in ALL, we established an MTX-resistant cell line (Reh-MTXR) from the human ALL Reh cell line by subjecting parental Reh cells to 10 cycles of a 24-h pulse of 200 nM MTX. Cell viability assays revealed that Reh-MTXR cells exhibited over twenty-fold resistance to MTX compared with Reh cells (Table 1; Figures 1A,B). Next, we assessed the drug sensitivities of Reh cells and Reh-MTXR cells to various chemotherapeutic drugs commonly used in clinical ALL treatment, including 6-mercaptopurine (6-MP), 6-thioguanine (6-TG), cytosine arabinoside (Ara-C), L-Asparaginase (L-ASP), vincristine (VCR), Daunorubicin, Idarubicin, and Etoposide. No significant differences were observed between Reh and Reh-MTXR cells in drug sensitivities to 6-MP, 6-TG, Ara-C, L-ASP, VCR, and Idarubicin. However, Reh-MTXR cells exhibited slight cross-resistance to Daunorubicin and Etoposide (Table 1 and Supplementary Figure S1).

**TABLE 1:** IC_50_ of chemotherapeutic drugs in Reh cells and Reh-MTXR cells.

Drugs	IC_50_ (nM) (mean ± SD)		Fold resistance
	Reh	Reh-MTXR	
MTX, 6h exposure	121.5 ± 13.7	2618.7 ± 118.5 ^****^	21.56
6-MP	379.6 ± 13.5	349.8 ± 27.2	0.92
6-TG	172.2 ± 5.6	155.4 ± 12.3	0.90
AraC	5.9 ± 0.3	6.9 ± 0.5	1.17
L-ASP (unit)	0.021 ± 0.002	0.019 ± 0.006	0.89
Vincristine	0.34 ± 0.04	0.32 ± 0.01	0.94
Daunorubicin	2.3 ± 0.2	3.4 ±0.1 ^***^	1.50
Idarubicin	1.3 ± 0.07	1.5 ± 0.1	1.18
Etoposide	47.9 ± 6.8	70.9 ± 0.5 ^**^	1.48

Values are presented as mean ± SD, of three independent tests.

**p < 0.01, ***p < 0.001, ****p < 0.0001, compared with Reh group, two-tailed Student’s t-test.

MTX exerts its cytotoxicity by blocking the synthesis of purines and thymine, leading to cell death through secondary genotoxic effects or apoptosis (Gonen and Assaraf, 2012). After a 6-hour MTX exposure, Reh and Reh-MTXR cells were cultured in a drug-free medium for an additional 48 or 72 h, and an apoptosis assay was conducted. Reh-MTXR cells showed a significantly reduced rate of MTX-induced apoptosis compared with Reh cells (Figures 1C,D). Moreover, no significant differences in cell proliferation were detected between Reh and Reh-MTXR cells (Figure 1E).

### 3.2 ABCG1 is upregulated in the MTX resistance cell line Reh-MTXR

**FIGURE 2:**
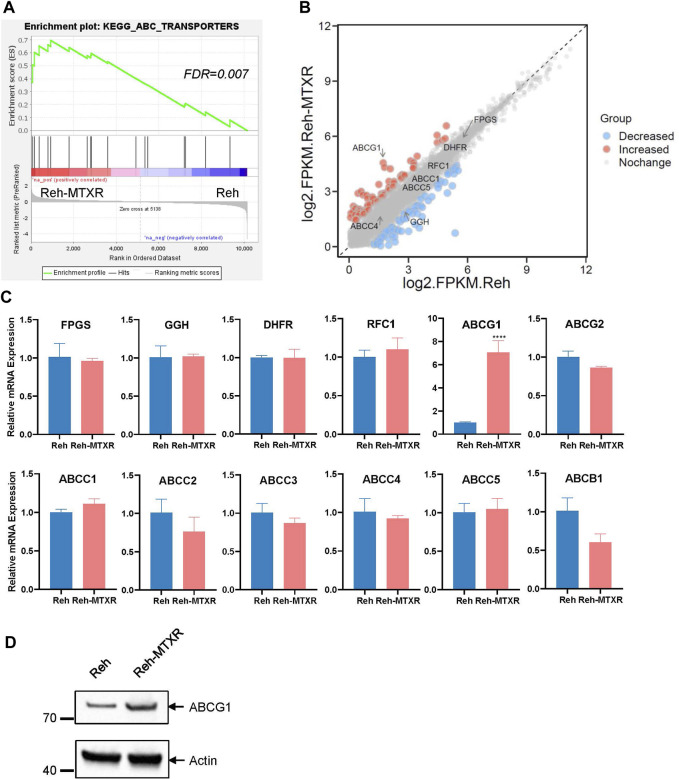
ABCG1 is upregulated in the MTX resistance cell line Reh-MTXR. **(A)** Illustration of the GSEA of upregulated ABC transporters pathway in Reh-MTXR cells. FDR <0.25 were defined as the significantly enriched gene sets. **(B)** RNA-seq analysis of differentially expressed genes upregulated and downregulated more than two folds in Reh cells and Reh-MTXR cells. Red dots represent upregulated genes, and blue dots represent downregulated genes. **(C)** mRNA levels of FPGS, GGH, DHFR, RFC1, ABCG1, ABCG2, ABCC1, ABCC2, ABCC3, ABCC4, ABCC5, and ABCB1 genes in Reh and Reh-MTXR cells were analyzed by RT-qPCR. Expression levels were normalized to a reference mRNA of Actin. Expression of genes in Reh was set as 1. Data are presented as mean ± SD of three independent tests, ****p < 0.0001, two-tailed Student’s t-test. **(D)** Cellular ABCG1 protein levels were detected by Western blot. Expression of Actin was measured as a loading control.

To comprehensively characterize potential differences between Reh parental cells and MTX-resistant cells, we conducted RNA sequencing on Reh and Reh-MTXR cells. Utilizing Gene Set Enrichment Analysis (GSEA), we identified an increased ABC transporters pathway in Reh-MTXR cells (Figure 2A). Nevertheless, the expression of ABCB1, ABCCs, and ABCG2 in the ABC transporter family and DHFR, FPGS, and GGH which were known to contribute to MTX resistance showed no differences between Reh and Reh-MTXR cells (Figures 2B,C and Supplementary Table S1). Interestingly, we observed a substantial increase in the expression of ABCG1 in Reh-MTXR cells compared to Reh cells (Figure 2B). These findings were further validated by RT-PCR (Figure 2C) and Western blot (Figure 2D). In conclusion, our results suggest the upregulation of ABCG1 in MTX-resistant cells.

### 3.3 Elevated ABCG1 is required for MTX resistance *in vitro* and *in vivo*

**FIGURE 3:**
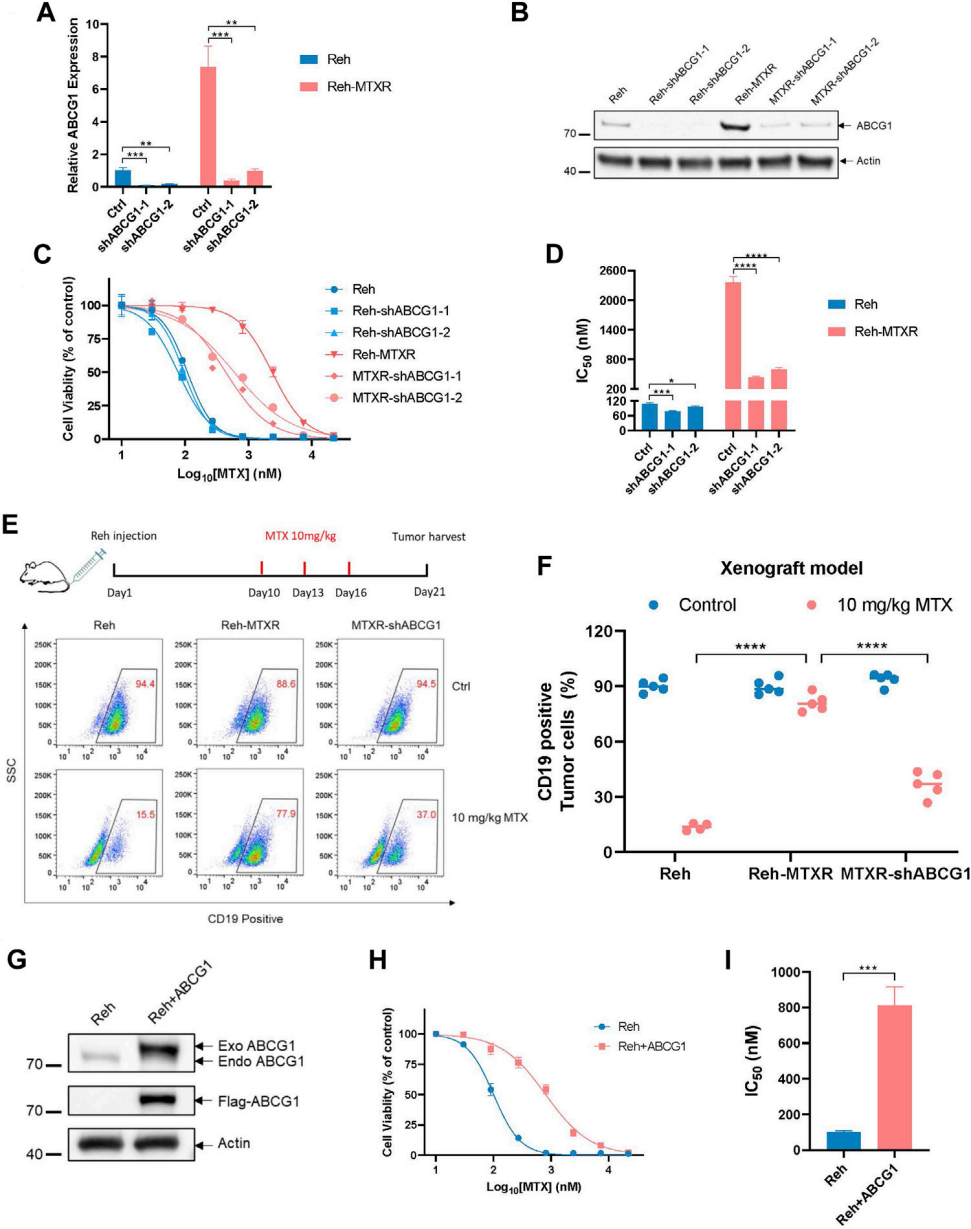
Elevated ABCG1 is required for MTX resistance *in vitro* and *in vivo*. **(A)** The analysis of ABCG1 knockdown efficiency in Reh and Reh-MTXR cells detected by RT-qPCR. The expression of ABCG1 in Reh was set as 1. Data are presented as mean ± SD of three independent tests, **p < 0.01, ***p < 0.001, two-tailed Student’s t-test. **(B)** ABCG1 knockdown evaluation in Reh and Reh-MTXR cells by Western blot. We measured the expression of Actin as a loading control. **(C, D)** Cell viability in response to MTX of various Reh cells. **(C)** Cell viability changes are shown as dose-dependent curves. Data are presented as mean ± SD of three independent tests. **(D)** IC_50_ values were obtained from experiments performed as in Figure 3C. ****p < 0.0001, two-tailed Student’s t-test. **(E, F)** Reh, Reh-MTXR and MTXR-shABCG1 cells were injected into the tail vein of B-NDG mice, and then treated with MTX as described in the upper schematic diagram. The ratio of CD19^+^ ALL cells in the bone marrow (BM) of the mice was detected by flow cytometric analysis. Data are presented as mean ± SD, n = 5 mice per group, ****p < 0.0001, two-tailed Student’s t-test. **(G)** ABCG1 is overexpressed in Reh cells. Expression of Actin was measured as a loading control. **(H, I)** Cell viability in response to MTX of Reh cells and ABCG1 overexpressed Reh cells. **(H)** Cell viability changes are shown as dose-dependent curves. Data are presented as mean ± SD of three independent tests. **(I)** IC_50_ values were obtained from experiments performed as in Figure 3G. ***p < 0.001, two-tailed Student’s t-test.

ABCG1, is an ABC transporter known for regulating cellular cholesterol and phospholipid homeostasis, but less explored in the context of ALL. To investigate whether the upregulation of ABCG1 contributes to the MTX resistance in ALL, we employed lentiviral shRNAs (Figures 3A,B) and the CRISPR-Cas9 system (Supplementary Figure S2A) to knock down ABCG1 in Reh and Reh-MTXR cells. The IC_50_ value of MTX slightly decreased in Reh cells and significantly decreased in Reh-MTXR cells upon ABCG1 knockdown (Figures 3C,D and Supplementary Figures S2B, C). Consistent with these findings, mice injected with Reh-MTXR cells exhibited MTX resistance compared to mice injected with parallel Reh cells, and ABCG1 knockdown effectively sensitized Reh-MTXR cells to MTX treatment *in vivo* (Figures 3E,F). Moreover, overexpression of ABCG1 in Reh cells conferred resistance to MTX (Figures 3G–I). These results indicate that the upregulation of ABCG1 is required for MTX resistance.

### 3.4 ABCG1 promotes MTX efflux in Reh-MTXR cells

**FIGURE 4:**
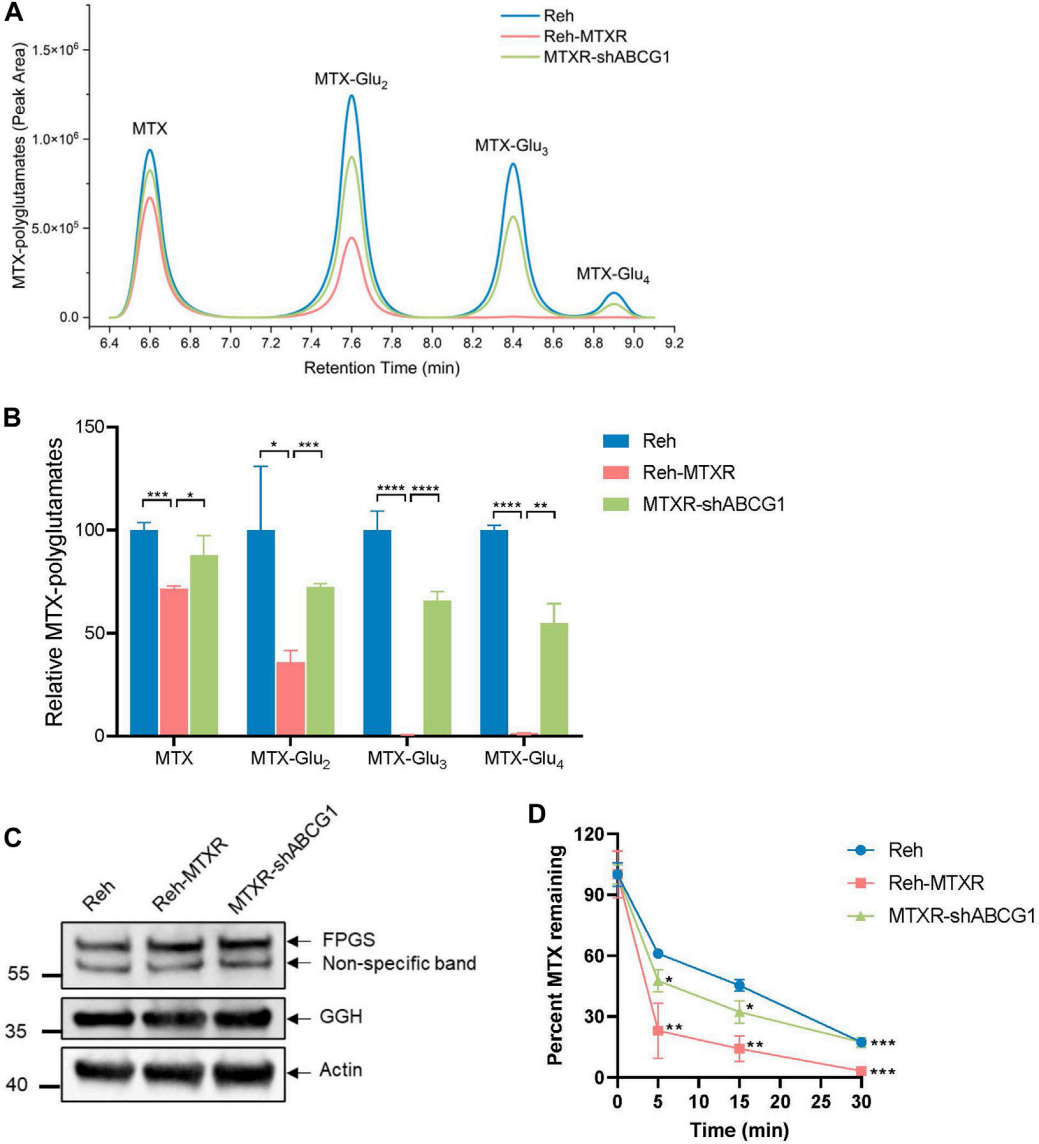
ABCG1 promotes MTX efflux in Reh-MTXR cells. **(A, B)** Reh, Reh-MTXR, and Reh-MTXR ABCG1 knockdown cells were exposed to 2 μM MTX, and after 24 h of accumulation of MTX, the levels of MTX polyglutamated metabolites were determined by LC-MS. **(A)** Diagram used for identifying MTX polyglutamated metabolites by LC-MS in various Reh cells. **(B)** Relative levels of MTX, MTX-Glu2, MTX-Glu3, and MTX-Glu4 in various Reh cells, each group was normalized to the Reh group, which was set as 100%. The experiment was performed in triplicates, and data are presented as mean ± SD. ***p < 0.001, ****p < 0.0001, two-tailed Student’s t-test. **(C)** Western blot analyses of FPGS and GGH in Reh, Reh-MTXR, and Reh-MTXR ABCG1 knockdown cells. We measured the expression of Actin as a loading control. **(D)** After 20 min of incubation in the medium containing 10 μM MTX, we washed the Reh, Reh-MTXR, and Reh-MTXR ABCG1 knockdown cells and incubated them in the drug-free medium. Efflux was measured by determining the level of MTX remaining in the cells after 0, 5, 15, and 30 min of incubation in the drug-free medium. The data are represented as the relative amount of MTX remaining in cells and are shown as mean ± SD. *p < 0.05, **p < 0.01, ***p < 0.001, two-tailed Student’s t-test, Reh-MTXR group compared with Reh group; MTXR-shABCG1 group compared with Reh-MTXR group.

Decreased antifolates polyglutamylation has been corroborated to cause MTX tolerance (Raz et al., 2016). Therefore, we assessed the levels of polyglutamylated forms of MTX in cells using liquid chromatography-mass spectrometry (LC-MS). As shown in Figures 4A,B, the amount of MTX and each MTX-Glu_2-4_ species significantly decreased in Reh-MTXR cells compared to Reh cells. Notably, knocking down ABCG1 in Reh-MTXR cells restored the accumulation of MTX and MTX-Glu_2-4_. The formation of MTX polyglutamates mainly relies on FPGS and GGH, while FPGS catalyzes glutamate addition, GGH removes glutamate residues. Thereby, reduced FPGS and/or increased GGH expression could decrease polyglutamylation of MTX. However, no significant differences in RNA or protein expression levels of FPGS and GGH were observed between Reh and Reh-MTXR cells (Figure 2C; Figure 4C), making it unlikely that the reduced formation of MTX polyglutamates in Reh-MTXR cells was due to changes in FPGS and GGH.

To further investigate whether the decreased accumulation of MTX in Reh-MTXR cells resulted from enhanced efflux, we employed a metabolic LC-MS method to monitor the MTX efflux. Cells were pretreated with MTX for 20 min, and then cultured in a drug-free medium for different times. As illustrated in Figure 4D, Reh-MTXR cells exhibited increased MTX efflux compared to Reh cells. Importantly, knocking down ABCG1 in Reh-MTXR cells significantly slowed MTX efflux. These results collectively indicate that ABCG1 accelerates MTX efflux, leading to decreased intracellular MTX and MTX polyglutamates levels, ultimately resulting in resistance to MTX.

### 3.5 ABCG1 inhibitor benzamil effectively sensitizes Reh-MTXR cells to MTX

**FIGURE 5:**
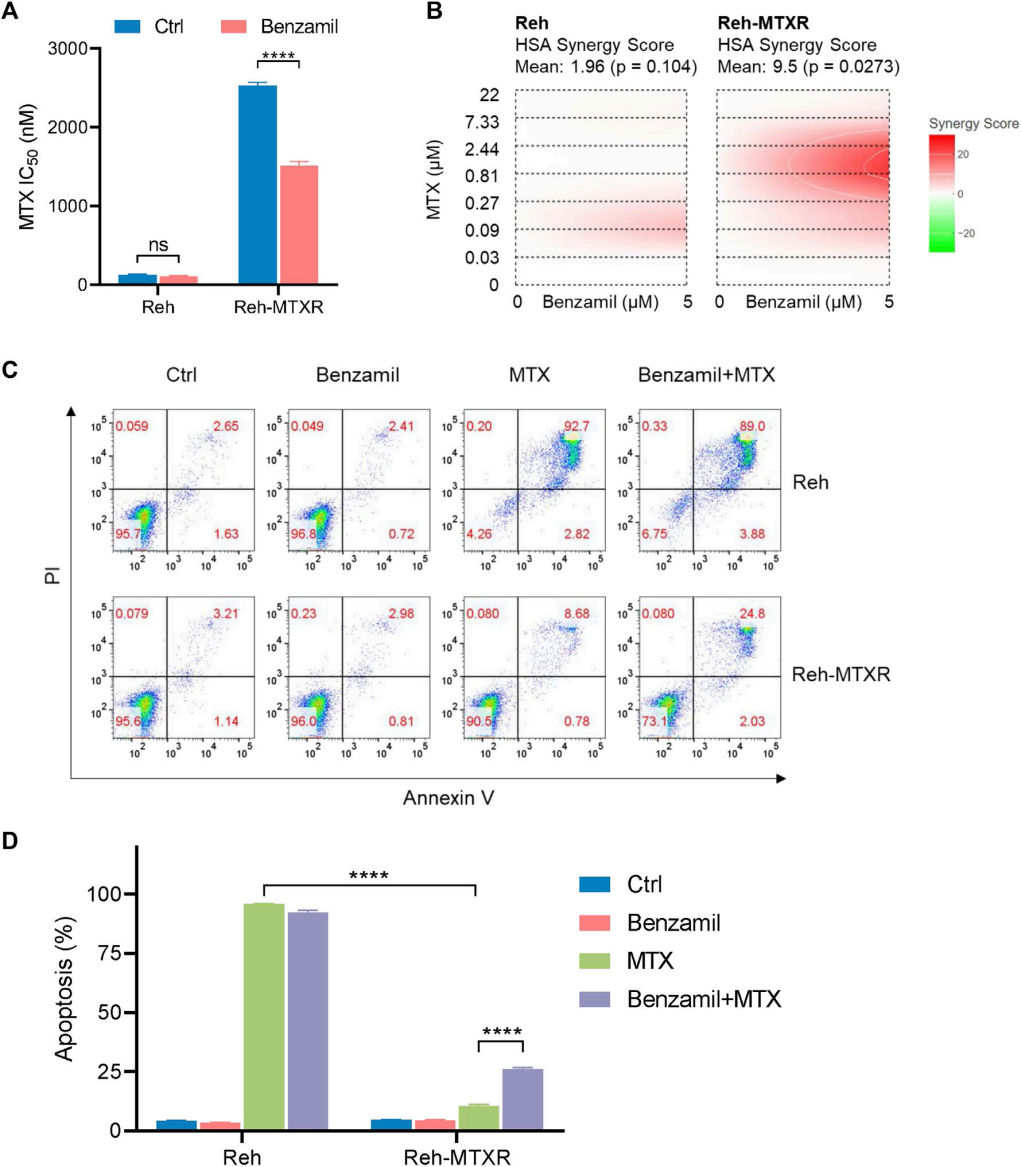
Benzamil effectively sensitizes MTX resistance in Reh-MTXR cells. **(A)** Various Reh cells were plated in the culture medium containing vehicle and 5 μM Benzamil separately; 1 day after plating, we exposed the cells to a gradient concentration of MTX for 6 h and then washed the samples with PBS. After that, cells were cultured with vehicle or 5 μM Benzamil for an additional 66 h. Cell viability was determined using the CellTiterGlo assay. IC_50_ values are shown as a bar graph. Data are presented as mean ± SD of three independent tests. ****p < 0.0001, two-tailed Student’s t-test. **(B)** Interactive analysis of MTX and Benzamil combination response using SynergyFinder. We calculated the degree of combination synergy using the HSA synergy scoring models. **(C, D)** Apoptosis levels of various Reh cells treated with vehicle, 5 μM Benzamil (72 h), 2 μM MTX (6 h MTX exposure of a 3-day growth) or both Benzamil (5 μM, 72 h) and MTX (2 μM, 6 h). Data are shown as mean ± SD of three independent tests, ****p < 0.0001, two-tailed Student’s t-test.

Our previous data have shown that upregulation of ABCG1 could lead to MTX resistance in ALL. We, therefore, postulated that targeting the function of ABCG1 may overcome MTX resistance in Reh-MTXR cells. Benzamil has been previously reported to inhibit ABCG1 (Cserepes et al., 2004; Skarda et al., 2021). Intriguingly, Benzamil treatment of Reh-MTXR cells could partially reverse the MTX resistance (Figure 5A). Calculation of the synergy score (Zheng et al., 2022) revealed that combining Benzamil and MTX synergistically suppressed survival in Reh-MTXR cells (Figure 5B). Conceivably, the results from flow cytometry also confirmed that Reh-MTXR cells treated with Benzamil could increase the MTX-induced apoptotic rates (Figures 5C,D). Our findings may present a potential approach for the future treatment of MTX-resistant ALL.

### 3.6 Upregulation of ABCG1 in Reh-MTXR cells is not induced by alterations in DNA methylation or histone acetylation

**FIGURE 6:**
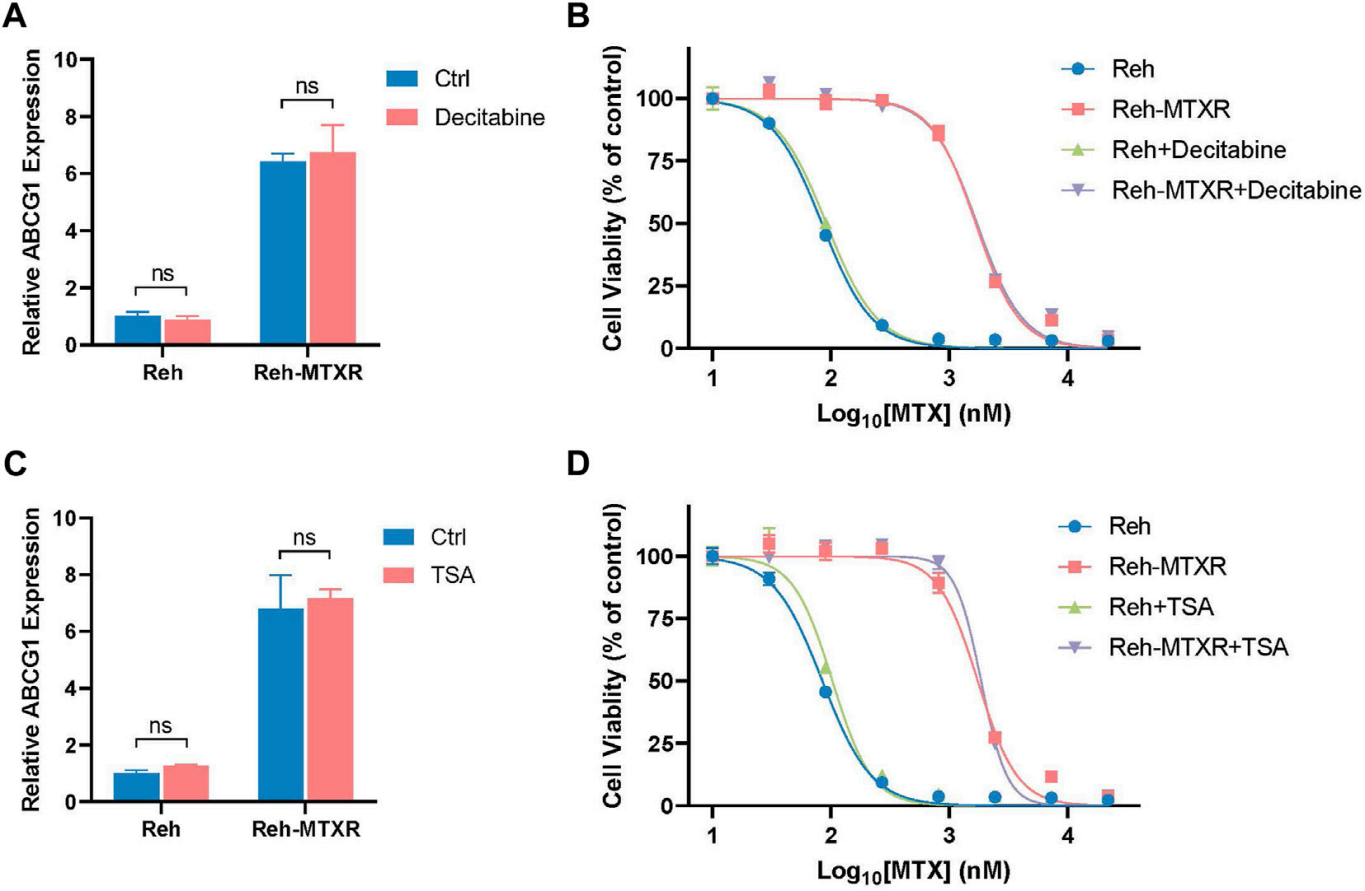
Upregulation of ABCG1 in Reh-MTXR cells is not induced by alterations in DNA methylation or histone acetylation. **(A and C)** Reh and Reh-MTXR cells were treated with 100 nM Decitabine **(A)** and 500 nM TSA **(C)** for 24 h, and levels of ABCG1 mRNA were analyzed by RT-qPCR. Expression levels were normalized to a reference mRNA of Actin. Expression of genes in Reh was set as 1. Data are presented as mean ± SD of three independent tests, ns *p* > 0.05, and two-tailed Student's t-test. **(B and D)** Reh and Reh-MTXR cells were treated with 1 nM Decitabine **(B)** and 50 nM TSA **(D)**, and cell viability in response to MTX of various Reh cells was measured. Data are shown as dose-dependent curves. Data are presented as mean ± SD of three independent tests and are indicated as a percentage of the control group, which was treated with vehicle only and set as the 100% control.

Numerous studies have elucidated that enhanced expression of ABC transporters, including ABCB1, ABCG2, and ABCC6, can be influenced by epigenetic modifications, specifically DNA methylation and histone acetylation, contributing to the development of acquired resistance to anticancer drugs (Wilting and Dannenberg, 2012; Adhikari et al., 2022). In light of this, we sought to determine whether the observed upregulation of ABCG1 in Reh-MTXR cells could be linked to alterations in DNA methylation or histone acetylation. To investigate this, we treated Reh and Reh-MTXR cells separately with the DNA methylation inhibitor Decitabine and the histone deacetylase inhibitor Trichostatin A (TSA), measuring the corresponding mRNA levels of ABCG1. Unexpectedly, neither Decitabine treatment nor TSA treatment exhibited significant effects on the ABCG1 mRNA levels in both Reh and Reh-MTXR cells (Figures 6A, C).

Simultaneously, we evaluated the impact of Decitabine and TSA on the viability of Reh and Reh-MTXR cells in response to MTX. Based on our results, Decitabine/TSA treatment demonstrated minimal effects on the cell viability of MTX in both Reh and Reh-MTXR cells (Figures 6B, D). These results suggest that the upregulation of ABCG1 in Reh-MTXR cells is unlikely to be attributed to alterations in DNA methylation or histone acetylation. This finding underscores the complexity of the regulatory mechanisms involved in ABCG1 expression and prompts further exploration to unveil the specific factors driving its upregulation in the context of MTX resistance.

## 4 Discussion

The resistance mechanisms associated with MTX are multifaceted and not yet fully elucidated. In this investigation, we employed the Reh human ALL cell line and its MTX-resistant counterpart, Reh-MTXR, developed through stepwise selection. Established theories propose various molecular pathways for MTX resistance, including impaired uptake due to downregulated RFC1, diminished FPGS expression, augmented GGH expression, and overexpression of DHFR or TS, among others (Fotoohi and Albertioni, 2008). Intriguingly, our study revealed that the classical mechanisms mentioned above may not be pivotal in Reh-MTXR cells, as no alterations were observed in RFC1, FPGS, GGH, DHFR, and TS expression. Instead, our focus turned to the upregulation of ABCG1 in Reh-MTXR cells, evident at both the mRNA and protein levels.

ABCG1 is a member of the ABC superfamily of membrane transporters (Liu, 2019), and this gene family has been implicated in drug resistance. While heightened levels of Pgp/ABCB1 have been associated with ALL relapse (Engle and Kumar, 2022), and overexpression of MRPs/ABCCs and BCRP/ABCG2 has been linked to resistance against various antifolates (Volk et al., 2002; Ansari et al., 2009; Liu et al., 2018; Jaramillo et al., 2019), our experiments found no significant differences in the expression of ABCB1, ABCCs, and ABCG2 between the Reh and Reh-MTXR cell lines. ABCG1, known for its role in cellular cholesterol and phospholipid homeostasis (Schmitz et al., 2001), has been linked to adverse prognoses in different tumors (Wang et al., 2016; Tian et al., 2017; Meng et al., 2021; Xi et al., 2022). However, to date, no reports have connected ABCG1 upregulation with MTX resistance. Our present study indicates that ABCG1 is upregulated in MTX-resistant cells, and silencing ABCG1 can reverse the MTX-resistant phenotype. To the best of our knowledge, this marks the first demonstration of increased ABCG1 expression in association with MTX resistance in a cell line. This discovery adds a novel dimension to the understanding of MTX resistance mechanisms and presents ABCG1 as a potential target for therapeutic intervention in MTX-resistant ALL.

MTX exhibits cytotoxicity through conversion to polyglutamylation forms, a process enhancing its cellular retention and the inhibition of target enzymes. Reduced formation of MTX polyglutamates is implicated in drug resistance. Our results demonstrate a diminished level of both MTX and MTX polyglutamates in Reh-MTXR cells. The balance between folylpolyglutamate synthetase (FPGS), catalyzing glutamylation, and γ-glutamyl hydrolase (GGH), removing glutamate residues, determines MTX polyglutamylation levels. (Raz et al., 2016). However, in Reh-MTXR cells, FPGS and GGH proteins are expressed at similar levels, suggesting unaltered functionality. Knocking down ABCG1 restores MTX and MTX polyglutamates accumulation, indicating its role in MTX resistance. MTX efflux is heightened in Reh-MTXR cells, and ABCG1 knockdown slows this efflux, implying that ABCG1 upregulation accelerates MTX efflux, leading to reduced MTX polyglutamates accumulation and, consequently, MTX resistance.

Given ABCG1’s role in MTX resistance, we hypothesized that targeting ABCG1 function could sensitize Reh-MTXR cells to MTX. Benzamil and taurocholate inhibit ABCG1 (Cserepes et al., 2004; Skarda et al., 2021), and our study reveals that benzamil treatment sensitizes Reh-MTXR cells to MTX, presenting a promising avenue for future ALL treatment.

Epigenetic modifications, such as DNA methylation and histone protein posttranslational modifications, are implicated in cancer development and drug resistance (Wilting and Dannenberg, 2012). Previous studies link ABC transporters (ABCB1, ABCG2, ABCC6) to DNA methylation and histone acetylation alterations (Wilting and Dannenberg, 2012; Zappe and Cichna-Markl, 2020; Adhikari et al., 2022). However, in this study, we find no evidence that DNA methylation or histone acetylation influences upregulated ABCG1 in Reh-MTXR cells. Further investigations are required to elucidate the underlying mechanisms.

In conclusion, our findings indicate that MTX resistance in Reh-MTXR cells results from ABCG1 upregulation, accelerating MTX efflux and diminishing MTX polyglutamates accumulation, and this upregulation is unrelated to DNA methylation or histone acetylation changes. Our study introduces the ABCG1 inhibitor benzamil as an effective means to sensitize Reh-MTXR cells to MTX, offering potential therapeutic strategies for MTX-resistant ALL.

## Data Availability

The datasets presented in this study can be found in online repositories. The names of the repository/repositories and accession number(s) can be found below: https://ngdc.cncb.ac.cn/gsa-human/, HRA005920.
