# Supplementary figures and images for: Up-regulation of ABCG1 is associated with methotrexate resistance in acute lymphoblastic leukemia cells
Source: Front Pharmacol. 2024 Jan 8;14:1331687. doi: 10.3389/fphar.2023.1331687 (PMC10800869; doi:10.3389/fphar.2023.1331687)

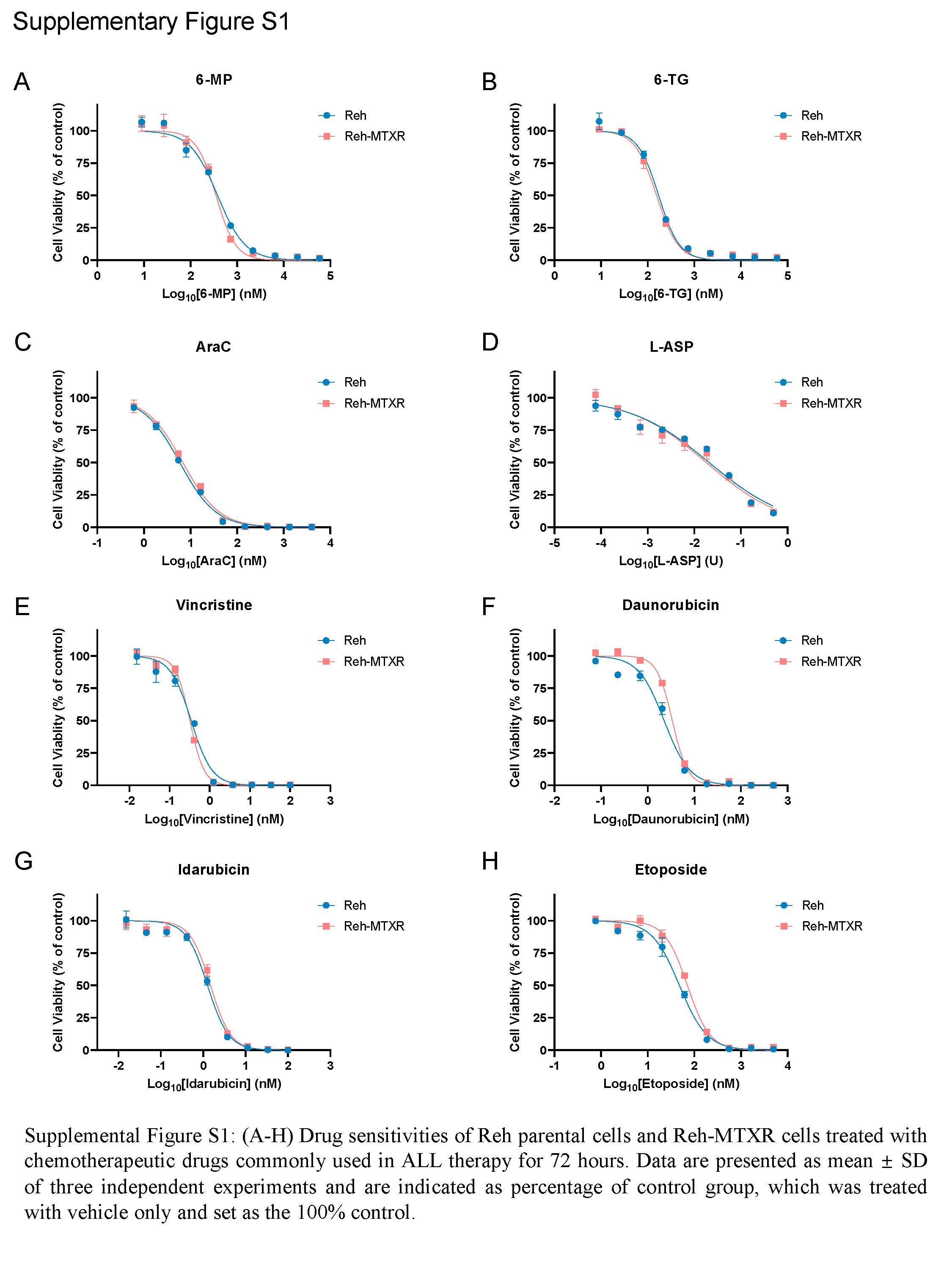

Supplement: Supplementary file 1 [file Image1.JPEG]

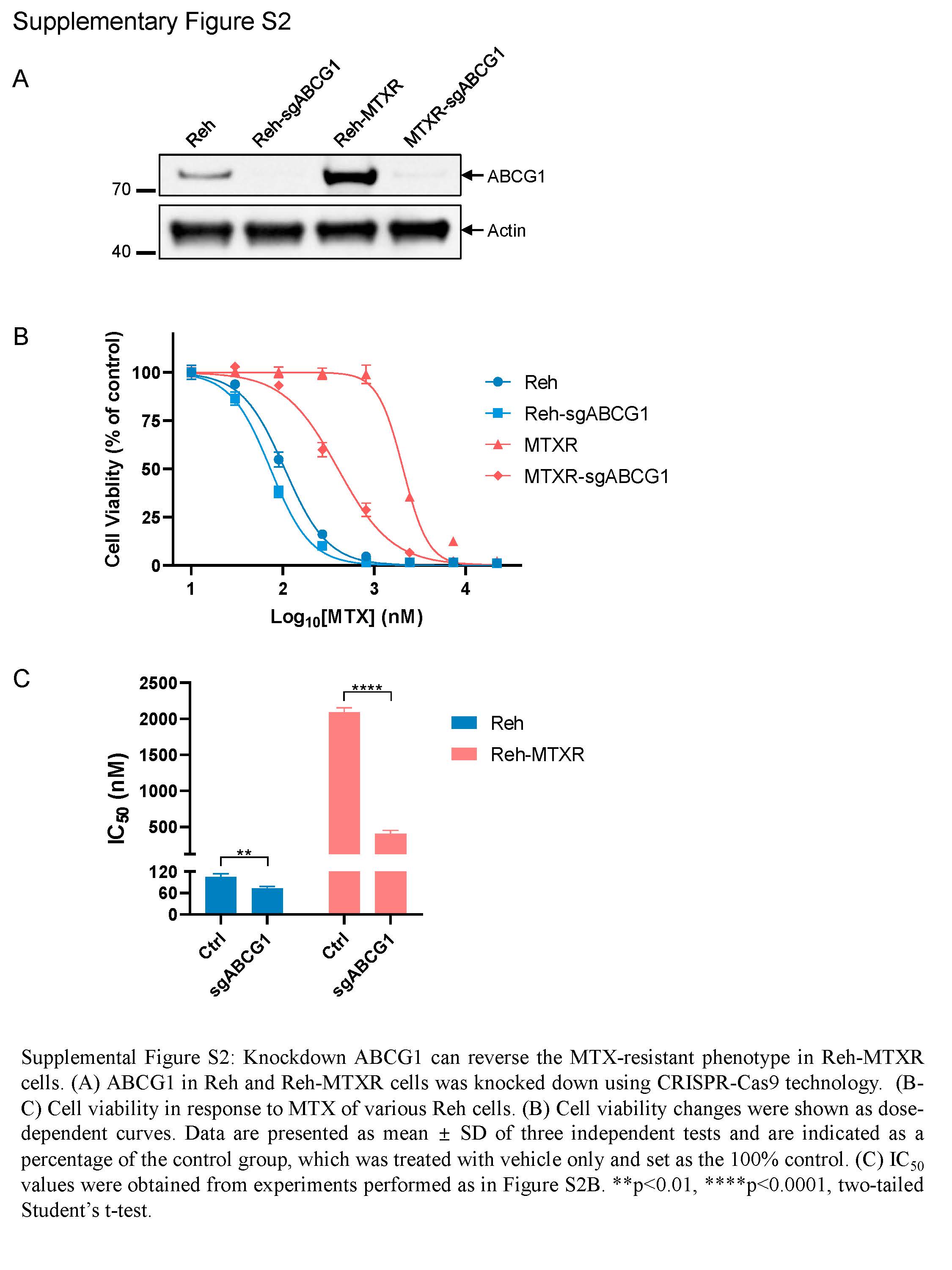

Supplement: Supplementary file 2 [file Image2.JPEG]
